# Radiomics based on lumbar CT to identify high-risk patients for OVCF in postmenopausal women

**DOI:** 10.3389/fragi.2025.1472060

**Published:** 2025-06-27

**Authors:** Qingsong Yu, Huangda An, Jiabao Chen, Aoran Ding, Zhe Lu, Haidong Wang, Lei Ma

**Affiliations:** Department of Spine Surgery, The Third Hospital of Hebei Medical University, Shijiazhuang, China

**Keywords:** OVCF, radiomics, osteoporosis, postmenopausal women, lumbar CT

## Abstract

**Objective:**

Osteoporosis vertebral compressive fracture (OVCF) is a severe complication in patients with osteoporosis. There were limitations in finding the risk factor of OVCF in previous evaluation techniques. In this study, we developed a radiomics model (R-model) based on a lumbar CT scan to identify vertebrae at high risk of OVCF in postmenopausal women.

**Method:**

Radiographic data of postmenopausal patients in our hospital from January 2021 to August 2022 were collected. All the patients received both dual-energy X-ray absorptiometry (DEXA) and lumbar CT scan. Images in a dataset of 329 vertebral bodies without compressive fracture in lumbar 1 to 4 were extracted by a 3D slicer and randomly divided into a training group (n = 230) and a test group (n = 99). A number of radiomics features (129) were automatically calculated by the pyradiomics module, and minimum-redundancy maximum-relevancy (mRMR), least absolute shrinkage, and selection operator (LASSO) were used to shrink features for R-model construction. The sensitivity, specificity, accuracy, and area under the receiver operating characteristic curve (AUC) of the T-scores and R-scores were calculated. The AUCs of the two models were compared using the DeLong test. Decision curve analysis (DCA) shows the clinical usefulness of the R-model.

**Results:**

Eight features were chosen to construct the R-model. The AUCs of the T-score and R-score in the training group were 0.845 and 0.945, respectively, and 0.818 and 0.914, respectively, in the test group. There was a significant difference (p < 0.001) between the AUCs of the two models, and the decision curve analysis (DCA) shows the R-model has a better overall net benefit than the T-score model.

**Conclusion:**

The radiomics model based on lumbar CT scans in postmenopausal women can identify and predict patients at high risk of OVCF with better sensitivity and accuracy than DEXA, even in patients with the same T-scores.

## Introduction

Osteoporosis is characterized by low bone mineral density (BMD) and micro-architectural deterioration of bone tissue that result in bone fragility (Sandhu and Hampson, 2011; Wang et al., 2021). It was reported that more than 80% of older patients with osteoporosis were postmenopausal women (Munoz et al., 2020). It was an occult disease and was usually first found by the occurrence of compressive fractures with severe complications (Sozen et al., 2017). The gold standard of diagnosis of osteoporosis was widely considered a T-score ≤ −2.5 standard deviations in the DEXA test (Kanis et al., 2019; Management of osteoporosis in postmenopausal, 2021). However, it has been found that T-scores could be easily disrupted by degenerative changes in vertebrae, such as spurs (Zou et al., 2019). Some scholars considered that a CT scan was a better screening tool for osteoporosis using the Hounsfield (HU), especially in patients with degeneration (Hocaoglu et al., 2021; Schreiber et al., 2011). However, the method for calculating HU values by using region of interest (ROI) in a limited number of axial pictures could also cause deviation.

Radiomics analysis, a new imaging analysis technique, provided a feasible and powerful tool for diagnosis by accurate analysis of image features (Lambin et al., 2017). Large numbers of features could be extracted from the image by using a radiomics analysis technique (Lambin et al., 2012). In oncology studies, the radiomics analysis technique was mainly used in diagnosis and evaluating prognosis (Liu et al., 2017). In recent studies, a model for predicting osteoporosis was established by using a radiomics analysis technique. Most of the studies were focused on screening for osteoporosis based on the diagnostic criterion of T ≤ −2.5 standard deviations in a DEXA test (Jiang et al., 2022; He et al., 2021). Until now, there was no study focused on predicting the value of OVCF in postmenopausal women based on radiomics analysis. In this study, radiomic features are extracted from the 3-D CT images of the entire lumbar vertebral body by using the radiomics analysis technique, and the purpose is to explore the value of predicting OVCF in postmenopausal women.

## Materials and methods

### Study population

This retrospective study was designed in accordance with the guidelines of the Declaration of Helsinki and was approved by the Institutional Review Board of our hospital. Documents of postmenopausal women patients who came to our hospital were collected from January 2022 to August 2022. The inclusion criteria were as follows: a. Postmenopausal women; b. Received lumbar CT scan and DEXA. c. An acute OVCF or no-fracture. Exclusion criteria: a. The time interval between DEXA test and lumbar CT was longer than 1 week. b. The thickness of the CT scan was greater than 1.25 mm c. History of lumbar internal fixation surgery. d. History of rheumatoid arthritis or long-term application of glucocorticoid drug. Most (145 of 182) patients met the criterion. Many (96) patients suffered from lumbar OVCF, and 49 patients did not. Parameters of the vertebral bodies from L1 to L4 in the lumbar CT scan were collected, except for the fractured vertebra. Parameters in the DEXA test were also collected. A total of 329 vertebral bodies were enrolled in this study and were divided by stratified random sampling into two groups: a training group with 230 vertebral bodies and a test group with 99 vertebral bodies.

### Image acquisition

Each patient underwent a lumbar CT scan using a helical 64-channel CT scanner (SOMATOM Definition AS plus 128, Germany). Acquisitions were performed in the helical mode with a tube voltage of 120 kVp, a tube current of 50–220 mA, and a slice thickness of 1.25 mm. The dual-energy X-ray absorptiometry (DEXA) scans were performed in each vertebral and hip with a DEXA system (Hologic Discovery, Hologic, MA, United States).

### Segmentation extraction

Two observers with more than 15 years of clinical experience in spinal surgery independently drew the vertebral body segmentation of the training and the test groups with a 3D slicer. The 3D slicer (version 5.0.3, https://www.slicer.org) is a free and open-access software platform for medical image processing (Fedorov et al., 2012). The functions of volume rendering and region of interest (ROI) were used to limit the extent of the vertebral body and exclude spinous processes and lamina. Tools of threshold, paint, and grow for the seed in segment editor procession were used to extract every vertebral body segmentation semi-automatically.

### Feature selection

All 329 vertebral body segmentations were used to extract radiomics features. A total of 129 features were obtained by applying the radiomics module to each segmentation. Eighteen features were excluded because they did not apply to all vertebral bodies, such as version and dimension The remaining 111 feature data elements were standardized by the z-score method, which retained all feature distribution characteristics but uniformized the order of magnitude of feature values. All features of training group segmentation were reduced in dimension to select the most predictive subset of features. First, the features with ICC>0.8 were selected. Then, minimum-redundancy maximum-relevancy (mRMR) was performed to eliminate redundant and irrelevant features. The most predictive subset of features was selected by using the least absolute shrinkage and selection operator (LASSO) regression model. Then, each predictive feature had a weighting coefficient, which was used to build the radiomics signature model (R-model).

### Model construction and evaluation

**FIGURE 1:**
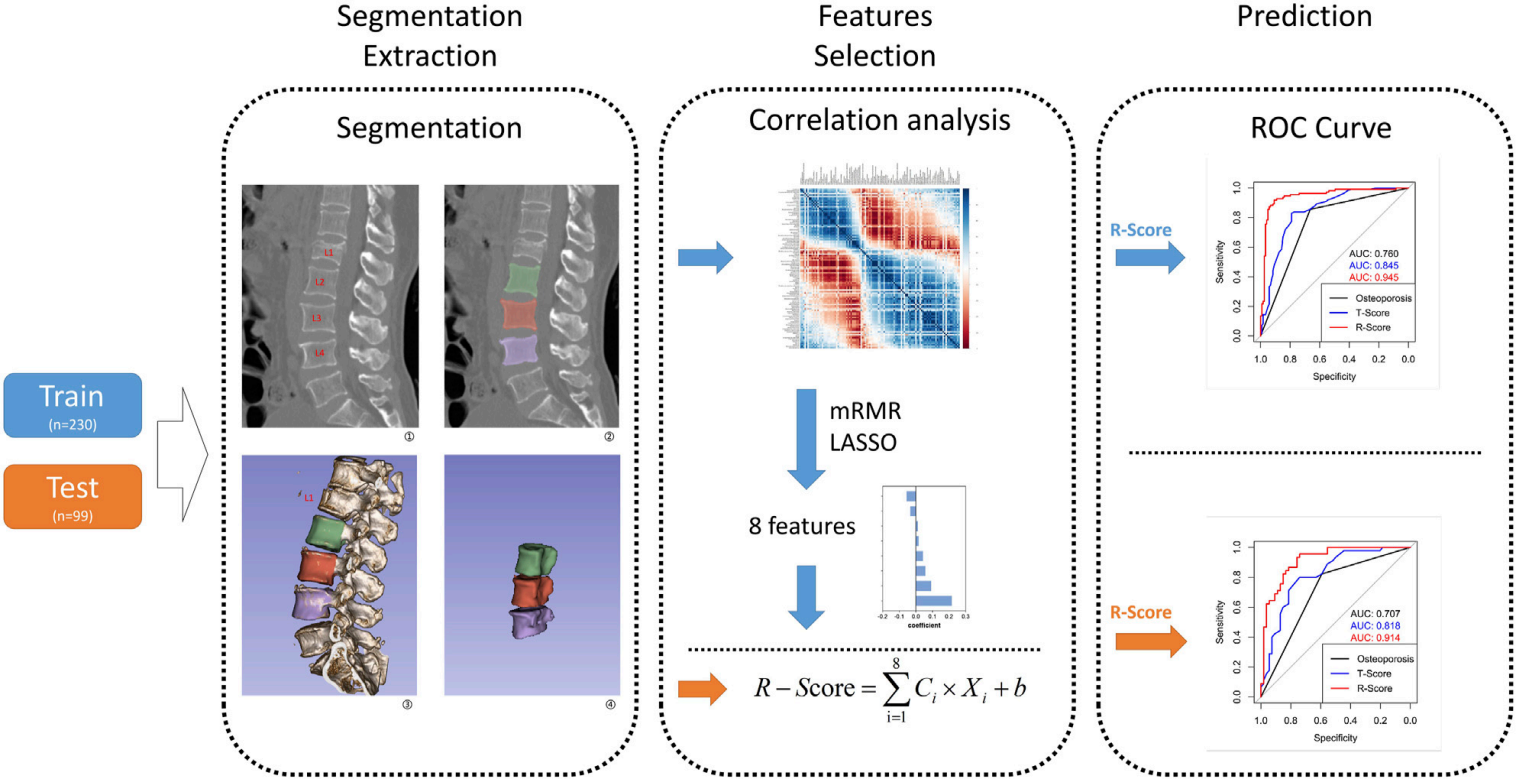
This flow graph illustrates the sequential process, starting from vertebra segmentation extraction to R-model construction and validation. The blue arrow represents the flow of the training group, while the orange arrow indicates the test group. In Step 1, segmentation extraction involves extracting a whole vertebra without attachments as features ROI. Step 2 entails selecting features from the training group to construct an R-model and calculate its corresponding R-score; meanwhile, features from the test group are directly used for calculating their respective R-scores. In Step 3, utilizing R-score, T-score, and osteoporosis criteria enables distinguishing between OVCF and NF patients in both groups, while also validating model consistency across both groups.

Using the predictive features selected from the training group, the R-model was constructed by the following formula. $R-S\text{core}=\sum_{i=1}^{n}C_{i}\times X_{i}+b$ . Ci represents the coefficient of features. Xi represents the value of predictive features after z-score conversion. b is the intercept of the LASSO result. The R-scores of all vertebral bodies in the training group and the test group were calculated to identify OVCF in the training group and verify OVCF in the test group. The results used the receiver operating characteristic (ROC) curve to demonstrate, and the AUCs were calculated for assessment. In the meantime, the T-scores of all selected vertebral bodies from both groups were recorded to establish another ROC curve for the T-model. Each vertebral body was diagnosed as osteoporosis or non-osteoporosis according to the standard of T-score ≤ −2.5. The diagnosis result was used to build an ROC curve to evaluate the clinical validation of R-score and T-score by the DeLong test. Finally, we used decision curve analysis (DCA) to evaluate the clinical utility of the R-model and T-model. The flow from feature selection to model construction and evaluation is demonstrated in Figure 1.

### Statistical analysis

Clinical continuous variables, including ages, weights, heights, BMI, T-scores of lumbar vertebrae, and T-scores of the femoral neck, were tested by using one-way analysis of variance (ANOVA) to compare between the training and the test groups. The chi-squared test was used to compare the diagnosis between the two groups. The z-score was used to transform the value of radiomics features. Those statistics steps above were calculated by using SPSS software (version 23.0; SPSS Inc., Chicago, IL, United States). The remaining statistical analyses for this study were performed with RStudio (Version: 2022.07.2 + 576, https://posit.co/products/open-source/rstudio/) based on R version 4.2.1, https://www/project.Org). The “mRMRe” and the “glmnet” packages in RStudio were used to process the mRMR and LASSO algorithms. It is worth mentioning that 25 prior features were set to be extracted by the mRMR algorithm, and the 25 prior features were shrunk to predictor features by LASSO regression. Ten-fold cross-validation was used to evaluate the predictive performance by choosing the suitable regular parameter λ. The “pROC” package was used to build the ROC curve, and DeLong`s test was used to compare the significance between the different ROCs. The “rmda” package was used to construct DCA for the test group. The “ggplot” package was used to print images of those plot results above. A *p* < 0.05 was considered statistically significant.

## Results

### Clinical characteristics

The clinical characteristics data are shown in Table 1. There were no significant differences in age, height, weight, BMI, T-Lumbar, or T-Neck between the training group and the test group.

**TABLE 1:** Clinical characteristics.

Characteristics	Training	Test	p
Diagnosis			0.718
OVCF	119	54	
NF	111	45	
Age	63.59 ± 9.860	62.72 ± 9.395	0.461
Height	155.21 ± 30.792	155.29 ± 32.406	0.983
Weight	63.16 ± 10.350	62.89 ± 9.689	0.824
BMI	25.27 ± 3.697	25.02 ± 3.730	0.598
T-Lumbar	−2.00 ± 1.644	−1.99 ± 1.530	0.962
T-Neck	−1.95 ± 1.185	−1.83 ± 1.227	0.400

Values are mean ± standard deviation; BMI is body mass index.

### Feature selection and model construction

**FIGURE 2:**
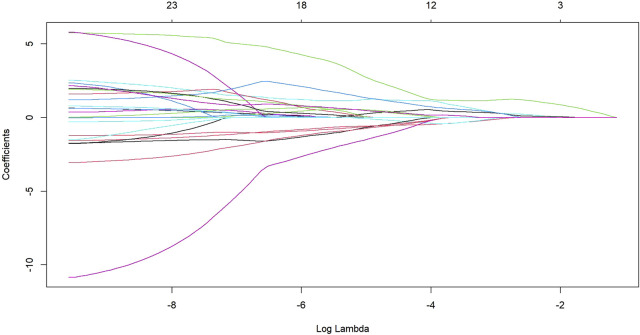
The LASSO regression showed the shrinkage of the 25 radiomics Zfeatures as log lambda increases.

**FIGURE 3:**
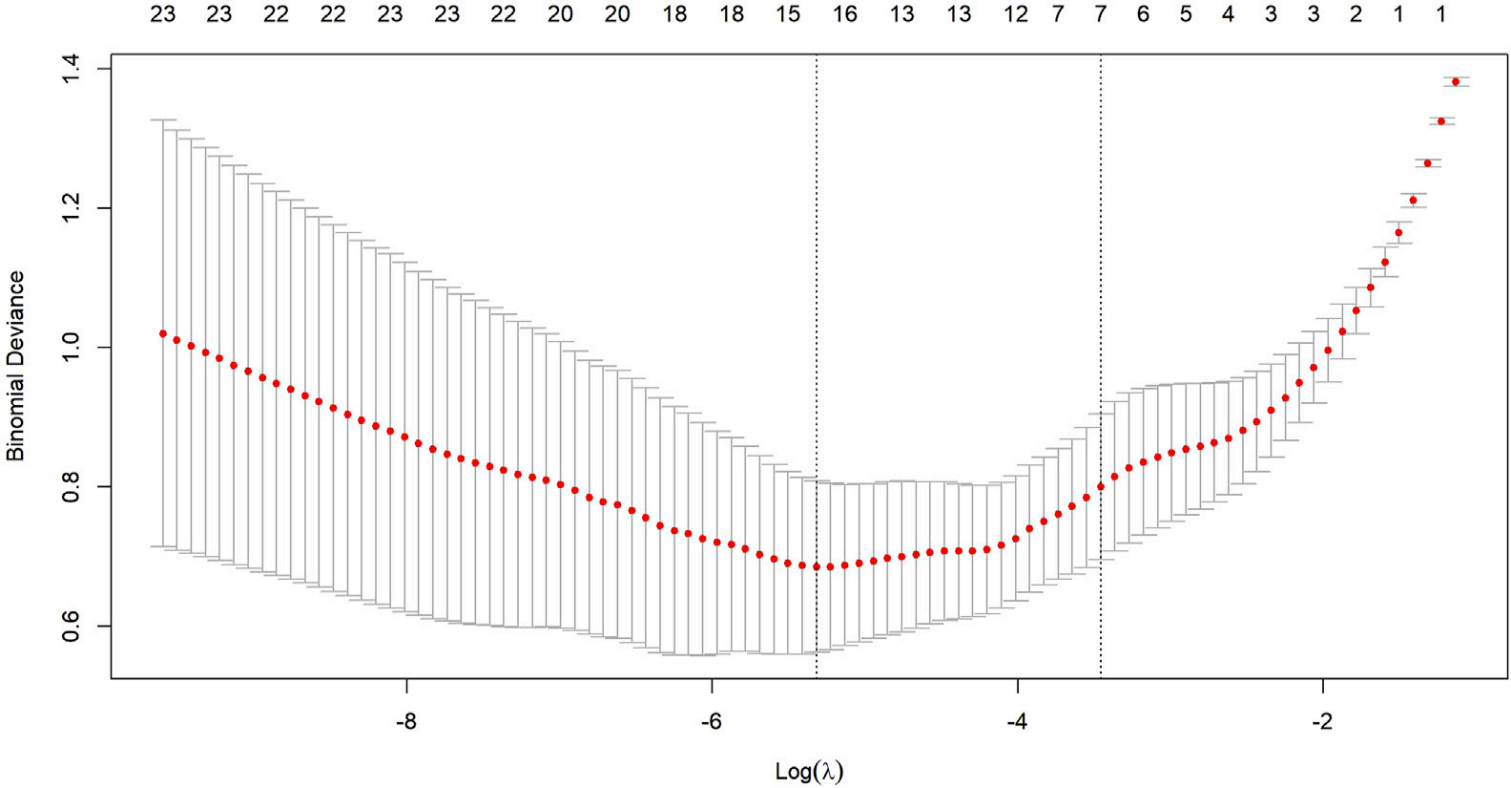
Ten-fold cross-validation is used to select an optimal lambda (lambda.min) for the LASSO model, ensuring a minimal number of Zfeatures while maintaining high accuracy.

**FIGURE 4:**
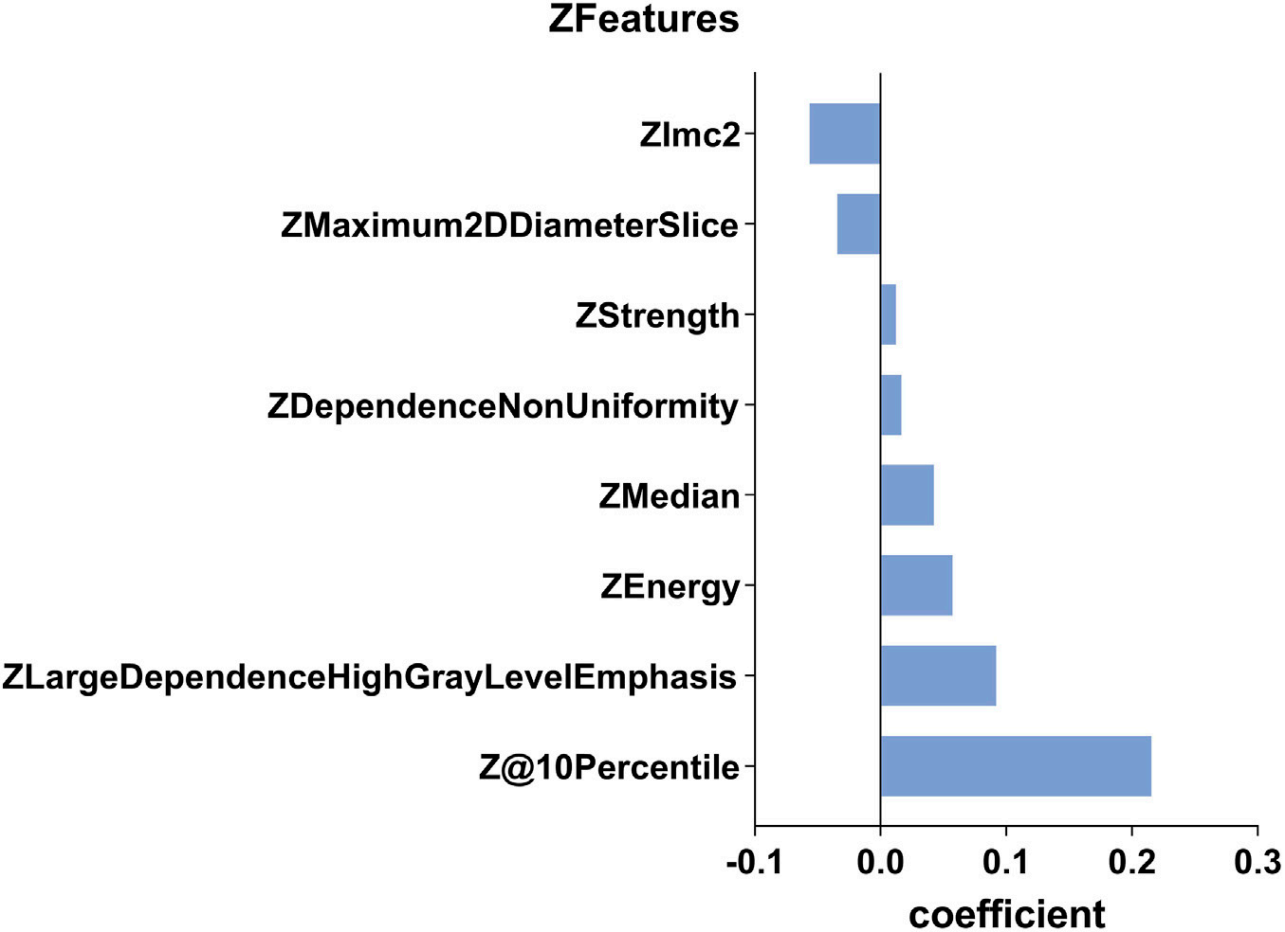
This histogram displays the coefficient of the eight predictive Zfeatures in the construction of the radiomics model. In the figure, the coefficients are sorted in the ascending order. A coefficient greater than 0 indicates a positive correlation, while the opposite suggests a negative correlation.

**FIGURE 5:**
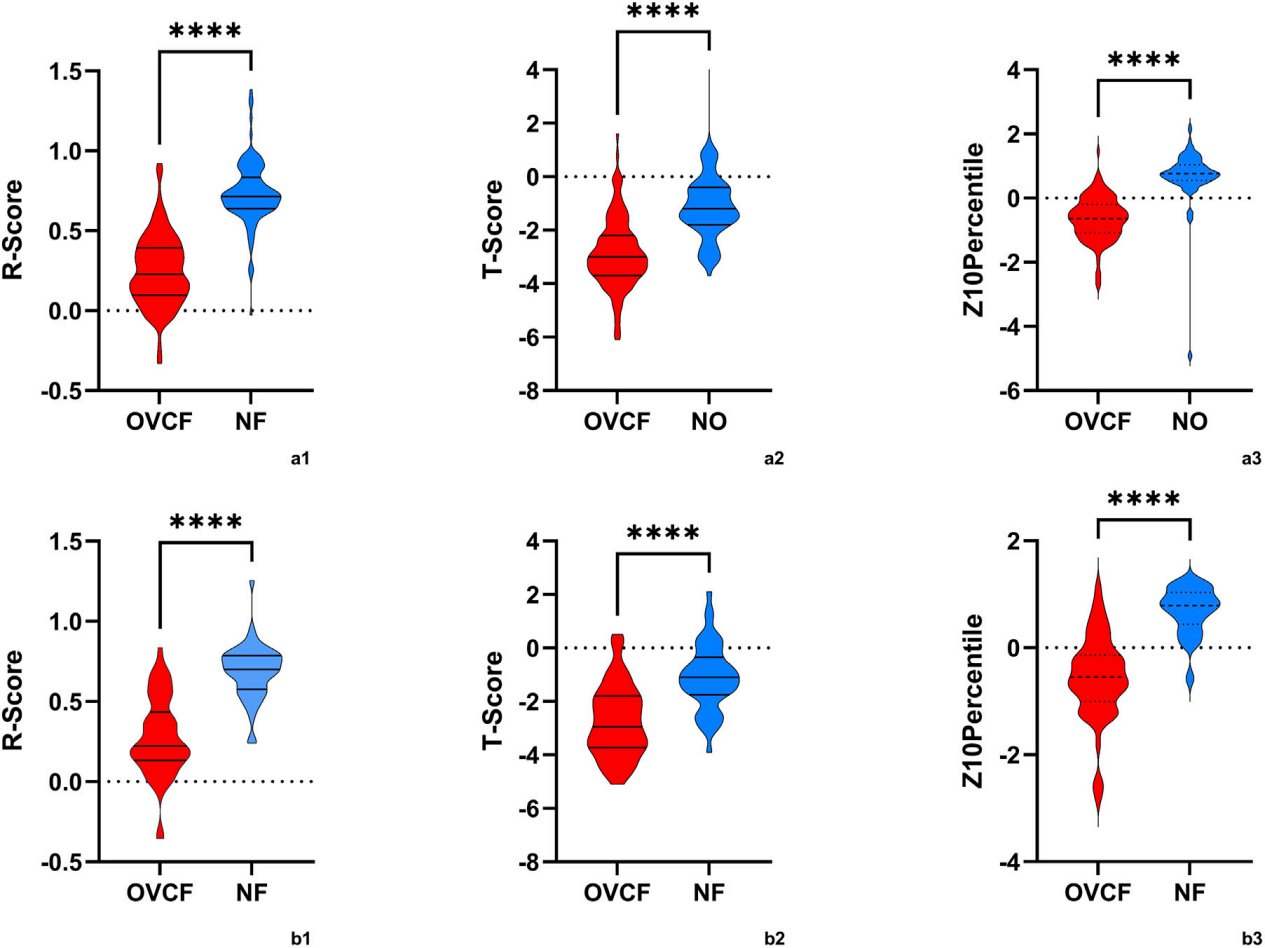
The violin plot presented the distribution of R-scores, T-scores, and Z10percentiles in OVCF patients and NF patients. (a1,2,3) is the training group, and (b1,2,3) is the test group. OVCF patients had substantially higher R-scores than non-OVCF patients in both training and test sets, indicating that the radiomics model assigns a significantly greater fracture risk to patients who experienced fractures. In contrast, the T-score distributions overlapped more between groups, reflecting why bone mineral density (BMD) alone is less discriminative. Median R-scores were higher in the OVCF group, as expected for a risk metric, while T-scores were generally lower (indicating osteoporosis) in the fracture group but with more overlap observed. These differences highlight the clinical advantage of the radiomics model in distinguishing between OVCF and non-OVCF patients.

After transforming the 111 features data using the Z-score method, the data were scaled down and zero-centered to a new data frame, with feature names changed to Z + Feature (e.g.,10 percentile was converted to Z10 percentile). Sixty-nine Zfeatures were selected based on an intraclass correlation coefficient (ICC) threshold of >0.8, ensuring inter-observer reliability. For the mRMR algorithm, we selected the top 25 features with maximum relevance and minimum redundancy to retain for LASSO regression (Figure 2). The LASSO regression was tuned using 10-fold cross-validation to select the regularization parameter (λ). The one-standard-error (1-SE) rule was applied to choose the final λ value of approximately 0.0315, which retained eight Zfeatures as the predictive features (Figure 3). The ICC threshold of >0.8 was chosen based on common practice in the literature to ensure feature stability, while the 1−SE rule was applied to favor a more parsimonious model (Figure 4). The R-scores of the two groups were calculated by the formula above and are shown in Figure 5. There was a significant difference in distribution between the OVCF and NF patients both in the training and the test group.

### Model performance evaluation

**FIGURE 6:**
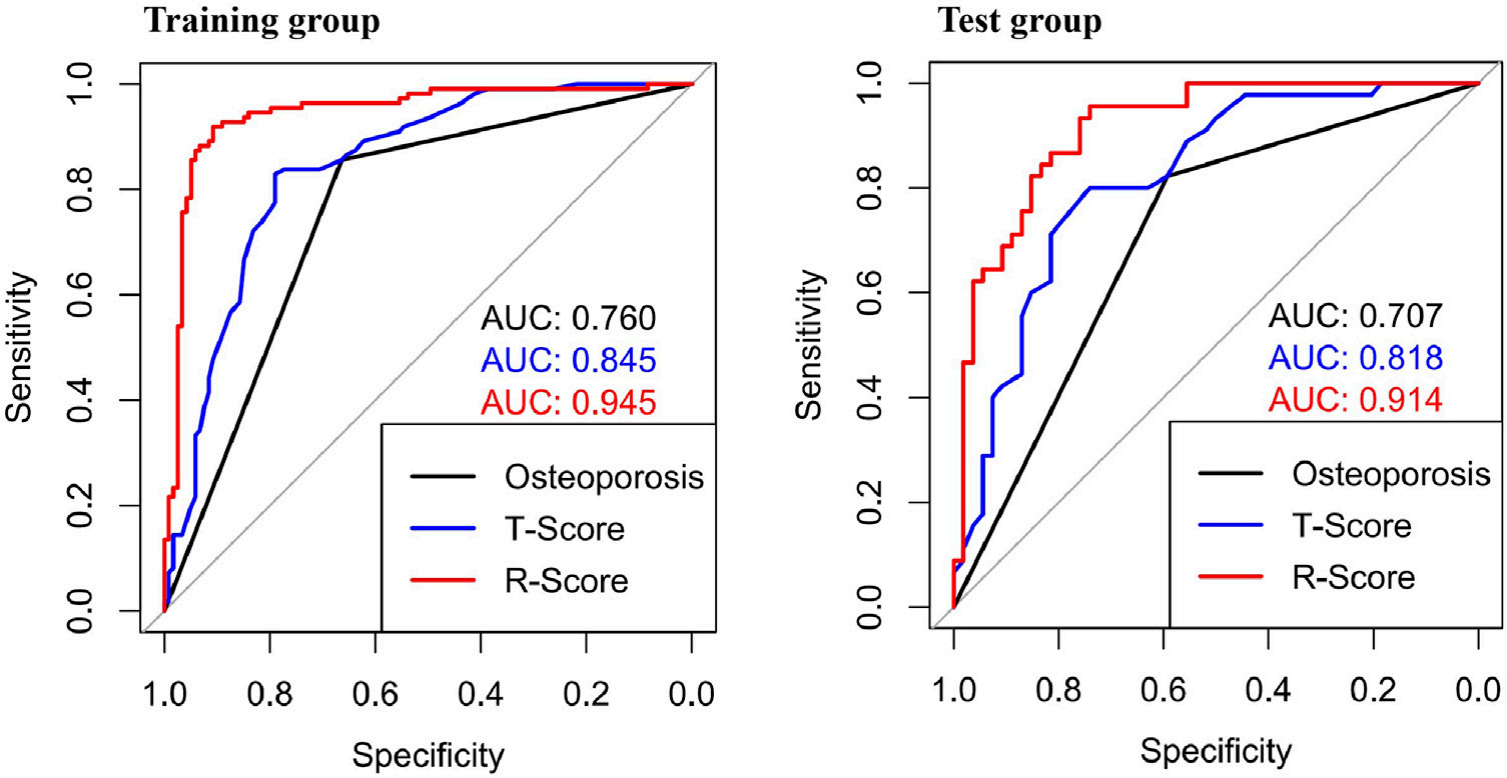
ROCs showed different AUCs in the training group and the test group. In both groups, the AUCs of the R-Score were higher than those of the traditional osteoporosis standard model and the T-Score.

**FIGURE 7:**
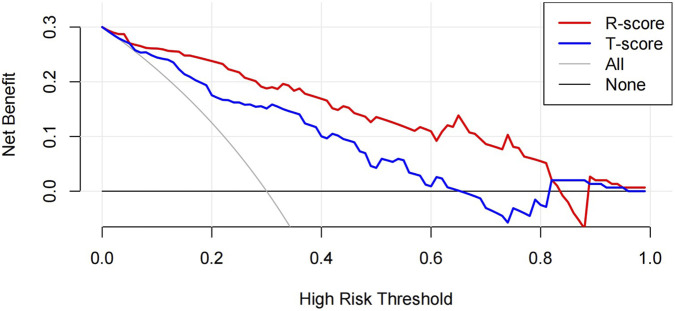
The DCA of the test group showed that the R-model provides a superior clinical benefit in predicting OVCF compared to the T-model.

The ROC curve was drawn with the R-score of each group, which was compared with the ROC curves of the vertebral body T-score and osteoporosis (Figure 6). The AUC of the R-score was 0.9454 (0.9133,0.9775) in the training group and 0.9144 (0.8593,0.9692) in the test group. There was a significant difference between the AUC of the R-score and the AUC of the T-score in the training group 0.8447 (0.7937,0.8957) by using DeLong`s test. A significant difference was also shown in the test group 0.8183 (0.7352,0.9015) (Table 2). Finally, DCA was used to describe the clinical usefulness of the R-model and the T-score. It indicated that the R-model had higher clinical benefits than the T-model (Figure 7).

**TABLE 2:** Diagnostic efficiency of different models in the training and the test group.

	Cut off	Accuracy%	Specificity%	Sensitivity%	AUC(95 %CI)	p-value of DeLong		p-value
						vs T-score	vs R-score	Training vs Test
Osteoporosis								0.3161
Training	0.5	76.3	66.4	85.6	0.7602 (0.6966, 0.8237)	<0.001	<0.001	
Test	0.5	71.8	59.3	82.2	0.7074 (0.6041, 0.8108)	<0.001	<0.001	
T-score								0.5982
Training	−2.05	81.0	79.0	82.9	0.8447 (0.7937, 0.8957)	-	<0.001	
Test	−1.85	77.3	74.1	80.0	0.8183 (0.7352, 0.9015)	-	0.01	
R-score								0.3398
Training	0.522	91.4	90.8	91.9	0.9454 (0.9133, 0.9775)	<0.001	-	
Test	0.402	85.8	74.1	95.6	0.9144 (0.8593, 0.9692)	0.01	-	

AUC, area under the curve; CI, confidence interval.

## Discussion

Osteoporosis is an occult disease without significant symptoms in the early stages until OVCF appears. Currently, the diagnostic gold standard for osteoporosis is a T-score of less than −2.5 in the DEXA test (World Health Organization, 1994). However, in clinical practice, we often encounter patients who suffer from OVCF with a T-score higher than −2.5. A study indicated that the DEXA pre-warning criteria for OVCF should increase to −1.7 (Chanplakorn et al., 2021). In our study, the risk of OVCF increased when the T-score was less than −1.85 in the training group. The sensitivity and specificity of the DEXA test could be affected by spurs and fat (Li et al., 2013). Previous research showed that two-thirds of lumbar vertebral bone mineral content (BMC) was contributed by posterior elements, and only one-third of the BMC came from the vertebral body (Wang et al., 2015). Those may explain why the BMC of the vertebral body would be poorer than the T-score indicated in the DEXA test. A Hounsfield unit (HU) score in a CT scan is regarded as one good detector for osteoporosis that is significantly correlated with both BMD and T-score (Schreiber et al., 2011; Choi et al., 2016). Zou et al. found that the HU value can be used to identify undiagnosed osteoporosis (Zou et al., 2019). However, there are some limitations to this method. The different parameters of CT always produce different HU values, and ROIs drawn by different people will lead to a difference in homogeneity. Quantitative CT (qCT) has been used to detect vertebral trabecular BMD with good sensitivity and specificity. However, this approach has problems with higher prices, additional radiation exposure, and the need for more specialized doctors (Cheng et al., 2021). Actually, the fracture-risk assessment tool (FRAX), which can more efficiently predict the occurrence of OVCF, has long been recommended to predict the probability of hip fracture and major osteoporotic fracture in the next 10 years (Johansson et al., 2017). However, due to the tedious clinical data and the lack of timeliness of prediction (Kanis et al., 2011), it has not been widely used by orthopedic surgeons. Therefore, a more accurate and timely way to predict the probability of OVCF is needed.

In the past 10 years, radiomics has developed rapidly. It was originally used for tumor identification, but now it has been used in multiple clinically aided diagnoses. Zhang H et al. used radiomics to detect brainstem infarction (Zhang et al., 2022). Kaviani P et al. used radiomics to differentiate the composition of kidney stones (Kaviani et al., 2022). There is no doubt that we have been inspired by some studies that verified radiomics through X-ray, CT, or MRI images to detect osteoporosis. Liu and Tang et al. used a radiomics model by MRI to predict new fractures in patients after percutaneous vertebral augmentation (Liu et al., 2022). Biamonte and Levi et al. verified that there was a significant difference in radiomics features between fragility fracture and no-fracture vertebral body (Biamonte et al., 2022). Hong, Dai et al. used radiomics based on abdominal CT images to predict the BMD of lumbar vertebrae (Dai et al., 2021). Hui, Yang et al. used radiomics by using an image of spinal CT to distinguish acute and chronic osteoporotic vertebral fractures (Yang et al., 2022). Rastegar S et al. used DEXA image features to improve the accuracy of identifying osteoporosis (Rastegar et al., 2020). Radiomics has shown a unique advantage for identifying bone differences in images. However, current AI algorithms for vertebral segmentation are not yet sufficiently accurate for fully automated use. Therefore, we employed a semi-automated segmentation approach using 3D Slicer, followed by manual review and modification by experienced clinicians to ensure accuracy. This method, while effective, is time-consuming. We anticipate that future advancements in AI technology will enable fully automated segmentation, thereby improving efficiency.

A radiomics model was developed in this study to identify high-risk vertebrae in a post-omics model and shows satisfactory results compared to DEXA in detecting patients with OVCF. The AUCs curve and DCA result verified that the radiomics model showed very good accuracy and sensitivity in detecting patients with a high risk of OVCF. The AUC of the R-score reaches 0.9454 (0.9133, 0.9775) in the training group and 0.9144 (0.8593, 0.9692) in the test group. It means that the radiomics model could reflect the degree of osteoporosis in the vertebral body and minimize interference factors. It is worth mentioning that this method does not rely on DEXA data and only uses data of the common lumbar spine CT scan, which is widely used in clinical practice. While clinical factors such as age and BMI were collected and analyzed (Table 1), no significant differences were found between the OVCF and control groups. This supports the robustness of our radiomics model, which focuses on imaging features to identify high-risk vertebrae. The intentional exclusion of these clinical factors from the model was to specifically highlight the value of imaging-based risk assessment.

Radiomics can easily extract thousands of image features, including one-dimensional, two-dimensional, and three-dimensional data, and fully exploit the potential of clinical images. Of course, it is necessary for radiomics to reduce the dimensionality and extract the subset of features that are most predictive. In this study, we use ICCs to eliminate some features with low coefficients. Next, we used mRMR to select 25 features with maximum relevance and minimum redundancy. Finally, we used LASSO regression to diminish some features with low representativeness. The remaining eight features were reserved to construct the R-model. Due to the Z-score change of features, the coefficient of the Zfeature could represent the feature’s effective strength. In OVCF patients, features of 10Percentile, Large Dependence High Gray Level Emphasis (LDHGLE), Energy, Median, Dependence Non-Uniformity (DN), and Strength had a higher expression, while Maximum 2D Diameter Slicer and Imc2 showed negative effects. The 10 Percentile and Median measured the different distribution of the gray-level intensity of CT image. Lower 10 Percentile and Median implied that the patients with OVCF had lost substantial BMC, especially in cancellous bone. The LDHGLE and DN described the gray change of image, the higher value of which may indicate more trabecula bone reserved. Higher Imc2 was found in OVCF patients, which meant that OVCF patients had a more complex texture than NF patients. We believed that the higher Imc2 may be associated with the heterogeneous changes in bone structure. However, a higher Maximum 2D Diameter Slicer appeared in OVCF patients, which indicated that the vertebral body with osteoporosis had a longer length in axial view. It may relate to degeneration and hyperplasia of vertebral bone.

It is worth noting that our radiomics model could complement existing fracture-risk assessment tools like FRAX. While FRAX incorporates clinical risk factors such as BMI, age, and nutrition-related factors, our CT-based radiomics model provides a purely imaging-based risk indicator. This could be integrated with clinical risk factors to improve overall risk stratification. For example, patients identified as high risk by our radiomics model might benefit from more aggressive nutritional supplementation (e.g., calcium, vitamin D, and protein) or pharmacotherapy, even if their BMD T-score alone would not qualify them for such interventions.

There are also some limitations of this study. First, this study was a single-center retrospective study with a limited sample size. The generalizability of the radiomics model to external cohorts is uncertain due to potential differences in CT scanner models or protocols, patient ethnicities, or osteoporosis management practices. While the internal hold-out test performance was strong, the robustness of the radiomics model to new data remains unproven without external cohort testing. Future validation in multi-center or independent populations is essential to confirm the model’s applicability. We acknowledge that the current study serves as an initial proof-of-concept model, and any claims of broad applicability should be tempered until further validation is conducted. Second, we regarded OVCF patients as the high-risk segment of the OVCF population. There is a deficiency of explanation for the likelihood of NF patients with a lower R-score developing a fracture in the future. Third, our study focused primarily on vertebral body changes in the images, neglecting the influence of age, BMI, muscle, physical performance, and other diseases on OVCF. Future research should further explore the integration of these clinical factors with imaging features to provide a more comprehensive assessment of fracture risk. Finally, the ROI was described manually and segmented semi-automatically, which was time-consuming and increased the risk of bias caused by individual cognitive differences. It is expected to be optimized and simplified by artificial intelligence in the future.

In conclusion, our study developed and validated a radiomics model based on lumbar CT scans in postmenopausal women, demonstrating better accuracy and sensitivity than DEXA in detecting the risk of OVCF. These findings are currently limited to this specific demographic.

## Data Availability

The raw data supporting the conclusions of this article will be made available by the authors, without undue reservation.
